# Typhoid Fever and Invasive Nontyphoid Salmonellosis, Malawi and South Africa

**DOI:** 10.3201/eid1609.100125

**Published:** 2010-09

**Authors:** Nicholas A. Feasey, Brett N. Archer, Robert S. Heyderman, Arvinda Sooka, Brigitte Dennis, Melita A. Gordon, Karen H. Keddy

**Affiliations:** Author affiliations: Malawi-Liverpool-Wellcome Trust Clinical Research Programme, Blantyre, Malawi (N.A. Feasey, R.S. Heyderman, B. Dennis);; National Institute for Communicable Diseases, Johannesburg, South Africa (B.N. Archer, A. Sooka, K.H. Keddy);; University of the Witwatersrand, Johannesburg (K.H. Keddy);; University of Liverpool, Liverpool, UK (M.A. Gordon)

**Keywords:** bacteria, enteric infections, Salmonella enterica, Salmonellosis, typhoid, Malawi, South Africa, epidemiology, case frequency, dispatch

## Abstract

To determine the prevalence of invasive nontyphoid salmonellosis and typhoid fever in Malawi and South Africa, we compared case frequency and patient age distribution. Invasive nontyphoid salmonellosis showed a clear bimodal age distribution; the infection developed in women at a younger age than in men. Case frequency for typhoid fever was lower than for salmonellosis.

Invasive nontyphoid salmonellosis (iNTS) was first described as an AIDS-related illness in Africa and the United States in the 1980s. Although incidence in industrialized countries declined, nontyphoid *Salmonella* (NTS) spp. serovars (predominantly *S. enterica* serovars Typhimurium and Enteritidis) remain a common cause of bloodstream and focal infection in sub-Saharan Africa for adults with HIV infection and children with HIV, malaria, and malnutrition. iNTS has a strong seasonal pattern in adults and children. In addition, epidemics of iNTS have been described as associated with the emergence of multidrug resistance in Malawi (*1*). Similarly, multidrug resistance is well recognized in iNTS in South Africa (www.nicd.ac.za/pubs/survbull/2010/CommDisBullMay10_Vol0802.pdf). Death rates are 20%–25% among adults and children (*1*). In sub-Saharan Africa, transmission is thought most likely to be between humans, and no food or animal source has been found, although epidemiologic data remain sparse (*2*).

In comparison to iNTS, *S. enterica* serovar Typhi is a highly adapted, invasive, human-restricted pathogen that in the 19th century caused considerable illness and death in the United States and Europe but now has the greatest impact in developing countries. In sub-Saharan Africa, perhaps surprisingly, typhoid fever is not associated with HIV among adults (*3*).

Regional data on the demography and prevalence of both iNTS and *S.* Typhi for sub-Saharan Africa are incomplete (*4*). Estimates of incidence of iNTS among children, 175–388/100,000 (*5**–**7*), and among adult HIV-prevalent cohorts, 2,000–8,500/100,000 (*8**–**10*), have been made separately, in different locations, giving no overall demographic picture. Estimates of the incidence of typhoid fever have relied on limited available data from sub-Saharan Africa (*11*). Although typhoid is usually regarded as an illness of school-age (>5 years of age) children and young adults, there is considerable heterogeneity; some sites in Asia report high incidences of typhoid fever among children <5 years of age (*12*). We compared case frequency and patient age distribution for the predominant types of invasive salmonellosis among febrile patients of all ages treated at our 2 centers in 2 regions in sub-Saharan Africa, Malawi and South Africa, before 2004.

## The Study

In Malawi, Queen Elizabeth Central Hospital is the government-funded hospital for Blantyre District, serving ≈1 million persons. From January 1998 through December 2004, persons from the community who came to the hospital with fever (adults >14 years, axillary temperature >37°C; and children <14 years and >1 month, temperature >37.5°C, and negative malaria test result) had venous blood taken for routine culture, as previously described (*1*). During 1998–2000, a manual culture system was used. From December 2000 onward, the same volume of blood was cultured by using the BacT/Alert 3D automated system (bioMérieux, Marcy l’Etoile, France). All isolates were identified by using standard diagnostic techniques. Outbreaks caused by individual NTS serovars were observed during this period, occurred simultaneously among adults and children, and showed an identical age distribution to baseline data (*1*).

In South Africa, active laboratory-based surveillance for *Salmonella* spp. was introduced nationally in 2003. Data from January 2003 through December 2004 were collected by the Enteric Disease Reference Unit, the national reference center representing data from >250 diagnostic laboratories across South Africa, serving ≈46 million persons (www.nhls.ac.za). Samples from normally sterile sites (bloodstream, cerebrospinal fluid, pleural fluid) from patients admitted to hospitals in South Africa were collected according to the clinician’s judgment, and isolates were submitted to the local diagnostic laboratory, where they were identified by using standard diagnostic techniques before being sent to Enteric Disease Reference Unit for confirmation of identification and serotype. No major outbreaks of typhoid fever or iNTS were observed during this period.

## Conclusions

In South Africa during 2003 and 2004, 1,318 cases of iNTS were microbiologically confirmed (67% *S*. Typhimurium, 10% *S*. Enteritidis, 7% *S.* Isangi, and 6% *S*. Dublin), and 105 cases of *S.* Typhi were identified under surveillance that included demographic data. In Malawi during 1998–2004, 62,778 blood samples were taken, of which 10,628 yielded pathogens. Information included 4,956 cases of iNTS (75% *S*. Typhimurium, 21% *S*. Enteritidis), for which demographic data were available for 4,044, and 105 cases of *S.* Typhi bacteremia, for which demographic data were available for 75.

**Figure 1:**
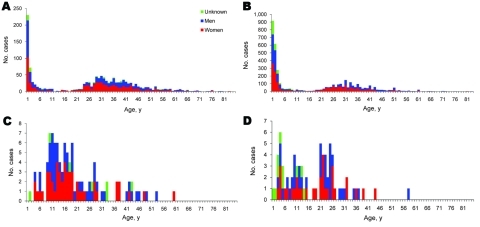
A) Age and gender distribution of patients with invasive nontyphoid *Salmonella* spp. infections in A) South Africa, 2003–2004, and B) Blantyre, Malawi, 1998–2004; and age and gender distribution of patients with *Salmonella enterica* serovar Typhi infection in C) South Africa, 2003–2004, and D) Blantyre, Malawi, 1998–2004.

Age distribution of patients with of iNTS and typhoid fever in Malawi and South Africa are shown in Figure 1. Despite the potential differences in sampling and surveillance intensity between sites, the data show a similar pattern of age distribution for iNTS at both sites, with a clear bimodal distribution. A peak was seen during the first 2 years of life, which rapidly declined thereafter until a second peak at ≈30 years. This age distribution was the same for all individual NTS serovars apart from *S.* Isangi and reflects the well-described risk factors for NTS infection: malaria, malnutrition, and HIV among children, and HIV among adults. A relative paucity of iNTS in the first few months of life has been reported from the Malawi center (*13*). We note that 32% (425/1,318) compared with 54% (2176/4044) of iNTS cases were in children <15 years of age for South Africa and Malawi, respectively. It is, however, not possible to say whether this observed difference may be explained by sampling bias, differences in population demographics, or iNTS specific risk factors in the 2 countries. Notably, patients from whom *S.* Isangi was isolated in South Africa were significantly younger (median age 5 years; p<0.001) than patients infected with other serovars.

**Figure 2:**
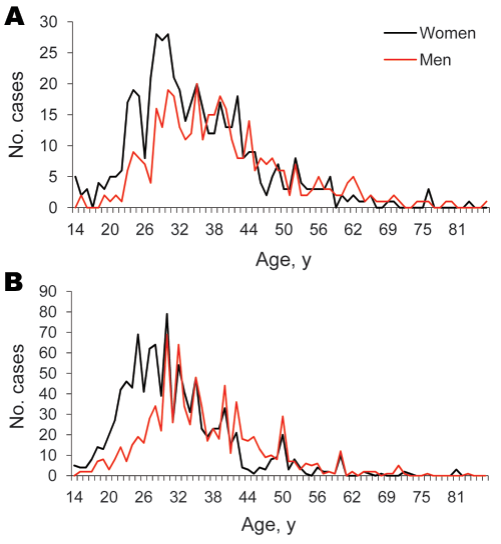
Age and gender distribution of adult patients (>14 years of age) with invasive nontyphoid *Salmonella* spp. infection in A) South Africa, 2003–2004, and B) Blantyre, Malawi, 1998–2004.

There was a significant gender difference in the age at which adults acquire iNTS (Figure 2). iNTS occurred in women at a younger age than men in South Africa (median age 30 years for women vs. 35 years for men; p<0.001) and Malawi (median age 33 years for women vs. 37 years for men; p<0.001). The principal risk factor for iNTS among adults is HIV (*3*), and this finding is consistent with the observation that women acquire HIV infection at a younger age than men. The HIV prevalence in those 15–24 years of age is 3× greater among women than men across sub-Saharan Africa. In South Africa the difference is even more marked, and HIV prevalence peaks in women 25–29 years of age and in men 30–34 years of age (*14*).

The relative frequency of iNTS cases detected by both centers was much higher than that of *S*. Typhi, which suggests a substantially higher number of cases. However, the different sampling protocols do not permit comment on absolute incidence rates.

Typhoid fever patients showed a markedly contrasting age distribution from that seen in iNTS, affecting mainly school-age children and younger adults, but differed slightly between the 2 sites. In Malawi, 15 of 75 typhoid cases were in preschool-age children, compared with only 5 of 105 of cases in South Africa. Again, the reasons for this cannot be determined.

It is noteworthy that the relative case frequencies and age distributions of iNTS and typhoid are so contrasting. Unlike in industrialized countries, NTS in sub-Saharan Africa is thought to be transmitted person to person, and a unique pathovar, *Salmonella* Typhimurium ST313, has emerged that shows genomic degradation similar to that seen in the human-restricted *S*. Typhi (*15*). Despite this evidence of convergent evolution, these data strongly suggest that the epidemiology and transmission routes of *S*. Typhi and NTS may be distinct. It remains to be seen what effects the wide availability of antiretroviral drugs or enhanced malaria eradication programs in sub-Saharan Africa will have on the demography of iNTS.
